# *Leucothoe eltoni* sp. n., a new species of commensal leucothoid amphipod from coral reefs in Raja Ampat, Indonesia (Crustacea, Amphipoda)

**DOI:** 10.3897/zookeys.518.9340

**Published:** 2015-08-25

**Authors:** James Darwin Thomas

**Affiliations:** 1 Reef Foundation Inc., Dania, Florida, USA

**Keywords:** Amphipods, coral reefs, ascidians, sponges, invasive species, model organisms, taxonomy

## Abstract

A new species of leucothoid amphipod, *Leucothoe eltoni* sp. n., is described from coral reefs in Raja Ampat, Indonesia where it inhabits the branchial chambers of solitary tunicates. With an inflated first gnathopod superficially resembling the genus *Paraleucothoe*, this new species has a two-articulate maxilla 1 palp characteristic of the genus *Leucothoe*. While described from coral reef environments in tropical Indonesia and the Philippines, it is an established invasive species in the Hawaiian Islands. The most likely mode of introduction was a US Navy dry dock transported to Pearl Harbor in 1992 from Subic Bay, Philippines.

## Introduction

While leucothoid amphipods are frequently encountered in marine faunal surveys and inventories information about their invertebrate hosts is rarely known and infrequently documented. The Leucothoidae, once thought to be a cosmopolitan and widespread taxon are now known to be a highly habitat-specific species complex. Taxonomic clarity within the group has been hampered by widespread and incorrect records of *Leucothoe spinicarpa* (Abildgaard, 1789). Upon further analysis many of these records are now proving to be discrete species, thus diminishing the cosmopolitan concept within the group (Krapp-Schickell and De Broyer 2014).

Because leucothoids lack a dispersive larval stage and frequently inhabit internal chambers of sessile invertebrate hosts they are potentially informative proxies for evolutionary diversity. Recent developments within the taxonomy of the Leucothoidae include: (1) availability of an electronic taxonomic database (Thomas and White 2014); and (2) widely deployed specialized *in-situ* underwater collecting methodologies. Specialized collection methods that isolate hosts and commensals together (sponges, ascidians, and bivalve mollusks) has led to re-examination of existing collections and spurred new efforts resulting in an increase in recently described leucothoid taxa for study and research (Myers 2013, Krapp-Schickel and DeBroyer 2014, Ortiz and Winfiled 2012, Thomas 1979, 1997a,b, Thomas and Klebba 2006, 2007, Thomas and Krapp-Schickel 2011, Thomas and Taylor 1981, White and Thomas 2009, White and Reimer 2012a, 2012b, 2012c, Winfield and Alvarez 2009, Winfield et al. 2009) including new approaches for molecular analysis (White 2010, 2011a,b, White and Reimer 2012d).

With their distinct morphology and common occurrence in shallow coastal marine environments, leucothoid amphipods drew the attention of early naturalists, resulting in some of the earliest recorded amphipod descriptions. While important taxonomic records, these early descriptions were often inadequately illustrated and described contributing to subsequent taxonomic confusion in the group. With increasing concerns about global climate change and loss of marine biodiversity, leucothoid amphipods are sensitive model organisms highly susceptible to a variety of toxicants and pollutants (Reish and Barnard 1969) and capable of providing a comparative diversity framework and serving as measures of change in marine ecosystems (Thomas 1993, 1997b). While the lack a larval stage limits widespread dispersal, some tube-dwelling and fouling community amphipods attain broad distributions as drifters on seaweed and algae, attached to floating debris, as components of fouling communities, and their occurrence in ballast water (Carlton 2010). Leucothoids, with their constrained distributions and commensal life history, can serve as valid indicators of environmental change and sentinels of lineage-based evolutionary history (Thomas 1997a). The use of leucothoids in such context depends on precise taxonomies and representative collections across broad marine habitats. This increased precision in determining composition and assessing threat levels is of interest especially for increasingly impacted coral reef systems (Thomas 1993, 1997b). Assessments incorporating cryptic biota could provide more refined and detailed insights than traditional coral reef inventories that incorporate organisms with large-scale dispersal capabilities (Hoeksema 2007).

Currently the revised Leucothoidae (*sensu stricto*) comprises 176 species in five genera. This includes 42 former anamixid species in *Anamixis* Stebbing, 1897 (23 spp.); *Nepanamixis* Thomas, 1997 (4 spp.); and *Paranamixis* Schellenberg, 1938 (15 spp.); and 134 leucothoid species comprised of *Leucothoe* Leach (132 spp.), and *Paraleucothoe* Stebbing, 1899 (2 spp.). Species in the former anamixid genera differ from other leucothoids in exhibiting radical sexual dimorphism, eusocial and harem guarding population structure, and tropic to warm temperate distributions. Species in *Leucothoe* and *Paraleucothoe* exhibit minor to moderate sexual dimorphism, and have tropic to polar distributions. Recent 18S rDNA sequence data by White and Reimer (2012d) suggest that the generic boundaries and definition of *Paraleucothoe* should be evaluated in the light of new molecular and morphological data.

## Materials and methods

Using SCUBA and specialized underwater collecting techniques amphipods were sampled *in-situ* from ascidians, sponges, and bivalves throughout Raja Ampat, Indonesia. Specimens were captured *in-situ* directly from their host either with a modified squirt bottle or by isolating hosts and substrata underwater in plastic bags and later coercing the amphipods from the host using a small amount of freshwater or formalin in the lab.

Specimens were either fixed in 2% buffered formalin or 70% ethanol. Prior to observation, specimens were gently cleaned with small sable hair brushes, and transferred to glycerin for dissection, illustration, and analysis. For SEM analysis, specimens were rehydrated to distilled water (three fluid changes for 10 minutes each), soaked in a dilute surfactant for 15 minutes (two drops of Tween 80 in 100 ml of water), briefly sonicated (10 seconds) to remove accumulated surface debris, and re-rinsed in distilled water (three fluid changes for 10 minutes each). This preparation protocol was modified from Felgenhauer (1987) by using a more finely graded alcohol series (5%, 10%, 15%, 25%, 35%, 50%, 60%, 70%, 75%, 80%, 85%, 90%, 95%, and 100%) to prevent distortion and shrinkage. Specimens were then fixed in salt water buffered osmium tetroxide (equal parts, under fume hood) for 2.5 hours, dehydrated in a graded alcohol series, transferred to acetone (three fluid changes for 10 minutes each), soaked in Hexamethyldisilazane Reagent (HMDS) for 15 minutes, air-dried overnight, and sputter coated with palladium for scanning electron microscopy. Photographs were taken with an ISI-DS-130 dual state scanning electron microscope.

## Results

*Figure Legend* – Capital letters in figures refer to the following appendages: A = antennae, Cx = coxae, E = epimera, Hd = head, LL = lower lip, Md = mandible, N = gnathopod, P = pereopod, T = telson, U = uropod, UL = upper lip, X = maxillae.

Capital letters to the right of each caption refer to the following: L = left, R = right. Lower case letters to the left of capital letters refer to the following adjectives: l = lateral, m = medial, x = magnified. Numbers to the right of capital letters refer to specific structures. “LW” in text refers to length/width ratios. Sexes are indicated by ♂ and ♀ symbols.

Material is deposited at the National Museum of Natural History, Leiden (RMNH) and at the Zoological Museum of Bogor (MZB)
Indonesian Institute of Sciences (LIPI). Additional material examined from the Bernice P. Bishop Museum (BPBM), Oahu, Hawaii, and the California Academy of Science, San Francisco, California (CASIZ).

### Leucothoe eltoni sp. n.

Taxon classificationAnimaliaAmphipodaLeucothoidae

http://zoobank.org/9C928A8A-EAC1-47E5-9A08-94AD9CC2D116

Figures 1 , 2 , 3 , 4 , 5 , 6 , 7 , 8 , 9

Paraleucothoe flindersi Stebbing, 1888, Muir 1997, pp 51–52

#### Type locality.

Reef slope, Yenweres Bay, Raja Ampat, Indonesia, 00° 29.216’S; 130° 40.394’E, coral reef slope, 20m.

#### Type material.

**Holotype.** Male A, 8.10mm; MZB Cru Amp 003, 10 December 2007, Yenweres Bay, Raja Ampat, Indonesia, 00°29.216’S; 130°40.394’E, JDT-RajAM-46, 20m, collected *in-situ* from branchial baskets of *Herdmania* sp. tunicates, James Thomas, collector.

**Paratypes.** Female B, 7.35mm; male C, 7.40mm; and six additional specimens. RMNH.Crus.A.5055, 10 December 2007, Station number JDT-RajAM-46, 20m, collected *in-situ* from branchial baskets of *Herdmania* sp. tunicates, James Thomas, collector.

#### Additional material examined.

Male and female specimens, RMNH.Crus.A.5056, 4 December 2004, Bunaken, Sulawesi, Indonesia, 1°37.063’ N; 124°46.966’ E. Station Indo04-01c, 8.5 m, from *Herdmania* sp. tunicates, reef wall in front of Living Colors Dive Resort. J. Thomas, K. White collectors. BPBM S11292-293, Pearl Harbor, Oahu, Hawaii, Station 6, 16 April 1996, from the sponge *Mycale grandis*, USN drydock “Machinist”. CASIZ 204559, Philippines, Batangas Province, Maricaban Island, Cemetery Beach, 13°41.063N; 120°49.813E, coral rubble, 5 m., from *Polycarpa* tunicate, J. Thomas, collector.

#### Etymology.

The specific epithet, eltoni, in honor of the rock musician Sir Elton John. Specifically, in reference to the large shoe-like first gnathopod of this species and the oversize boots Elton John wore as the local pinball champion in the movie “Tommy” (1975).

#### Diagnosis.

Male holotype A. Antenna 1 and 2 short, less than 0.10 body length; maxilliped, inner margin of outer plate crenulate, palp 2-articulate; gnathopod 1, carpus and propodus greatly enlarged; carpus setose posteriorly; distal margin of propodus tumid, inflated; gnathopod 2, palm oblique with 3 concavities separated by truncate projections; pereopods 5-7, article 4 extending beyond 0.5× of article 5.

#### Description of male holotype A.

Ratios of antenna 1 and 2, 0.10 and 0.09 body length; relative lengths of antenna 1 and 2, 1.00:0.89, flagellae 8 and 6-segmented. Anterior margin of head broadly truncate; mid-ventral keel produced, anterior margin produced dorsally as small knob, tapering posteriorly, ventral margin straight.

**Coxae.** Coxae 1-4 width ratios, 1.00:1.87:1.37:1.40, coxa 4 posterior margin widest mid posteriorly, tapering proximally, coxa 5-6 bilobed; coxa 7 reduced, ovate.

**Upper lip.** Asymmetrically lobate, anterior margin setose.

**Mandibles.** Both lacking molars; palp 3-articulate, ratio of articles 1-3 1.00:2.50:2.60; incisors moderately dentate. Left mandible, palp articles 2-3 with 2 anterior and 2 apical setae; lacinia mobilis large, strongly toothed; 13 raker spines, two distal raker spines enlarged and modified. Right mandible, palp articles 2-3 with 13 anterior and 2 apical setae; lacinia mobilis an elongated flake; 15 raker spines.

**Maxillae.** 1, palp 2-articulate with four apical setae, and two rows nine and eleven facial setae; outer plate with seven apical setae and nine facial setae; inner plate small, ovate, with single apical seta. Maxilla 2: inner plate, distal margin with 6 apical setae and 6 submarginal setae, 20+ facial setae; outer plate with 5 marginal medial setae and 19 facial setae.

**Maxilliped.** Inner and outer plates reduced; inner plates fused, with three stout apical setae and numerous fine facial setae; outer plate, anterior one third of medial margin tuberculate; palp article 1 with several apicodistal setae on medial dorsal margin and numerous marginal setae on ventral margin; article 4 with dense row of oblique and marginal setae on both dorsal and ventral margins; article 3 apical margin and dactyl with dense covering of pubescent setae.

**Gnathopod 1.** Coxa lobate, LW 1.25; basis linear, LW 3.66, anterior margin serrate with 22 long setae and single posterodistal apical seta; carpus expanded, basally stout, recurved distally with sharp apex; posterior margin with approximately 49 long recurved setae along 0.18-0.94 of carpal margin and 12 short, submarginal mediofacial setae; propodus, anterior margin greatly inflated, circular, LW 1.50, posterior margin expanded, with approximately 10 short posterior setae; dactyl reduced, straight, closing medially in groove on propodus.

**Gnathopod 2.** Coxa oval, expanded distally, distal margin smooth, LW 0.87; article 2 linear, LW 4.00, with tuft of six long posterodistal setae; carpal lobe slender, reaching 0.32 along propodus, distal margin expanded and subtruncate, lateral margin serrate, anterior margin oblique, with 15 rows of 6-15 medial setae; propodus, palm oblique, LW 4.25 with three major and two minor projections and two major and two minor concavities, primary mediofacial setal row extending 0.76 of propodus, secondary setal row extending along posterior margin, thicker proximally; dactyl smooth, gently curved, reaching 0.70 of propodus.

**Pereopods 3-4.** Pereopod 3, coxa elongate ventrally, LW 1.47; basis elongate, anterodistal margin slightly produced, posterior margin with 6 submarginal setae, LW measured at midpoint 6.61; Pereopod 4, coxa distal margin rounded, ventral and posterior margins slightly excavate, posterior margin serrate with 9 small submarginal setae, LW 1.13.

**Pereopods 5-7.** Coxae of 5-6 bilobed; coxa 7 small, ventrally convex; pereopods 5-7 bases moderately expanded, LW 1.42:1.20:1.09, posterior margin 5-6 smooth, 7 serrate; pereopods 5-7 article four with extended posteroventral lobe reaching 0.92:0.75:0.66 of article 4.

**Epimera 1-3.** Ventral setae 8:4:3, posterior margins round.

**Uropods 1-3.** Relative uropod lengths, 1.00: 0.75: 0.77; relative lengths of peduncles 1-3, 1.00:0.84: 0.85; uropod 1, peduncle 1.07 rami length, with 11 medial and 4 lateral setae; outer ramus subequal to inner ramus, with 12 lateral and 0 medial setae, margins minutely crenulate; inner ramus with 4 medial and 5 lateral setae, margins of rami minutely crenulate; uropod 2 peduncle 0.81 rami, with 0 medial and 2 lateral setae; outer ramus 1.30 inner ramus, with 0 medial and 4 lateral marginal setae; outer ramus with 2 medial and 4 lateral marginal setae; margins of rami minutely crenulate; uropod 3, peduncle 0.93 rami, with 1 lateral apical and 5 medial setae; outer ramus 1.34 inner ramus; with 2 medial and 3 lateral marginal setae; outer ramus with 0 medial and 4 lateral marginal setae, margins of rami minutely crenulate.

**Telson.** LW 1.95, apical margin minutely tridentate, with 2 apical and two pairs of 2 facial setae.

#### Description of female paratype B

, 7.35 mm. Similar to males except for gnathopods 1 and 2. Gnathopod 1, carpus and propodus not greatly inflated, propodus slightly swollen distally. Gnathopod 2, palm of propodus lacking distinct tuberculation and concavities found in males.

**Figure 1.**
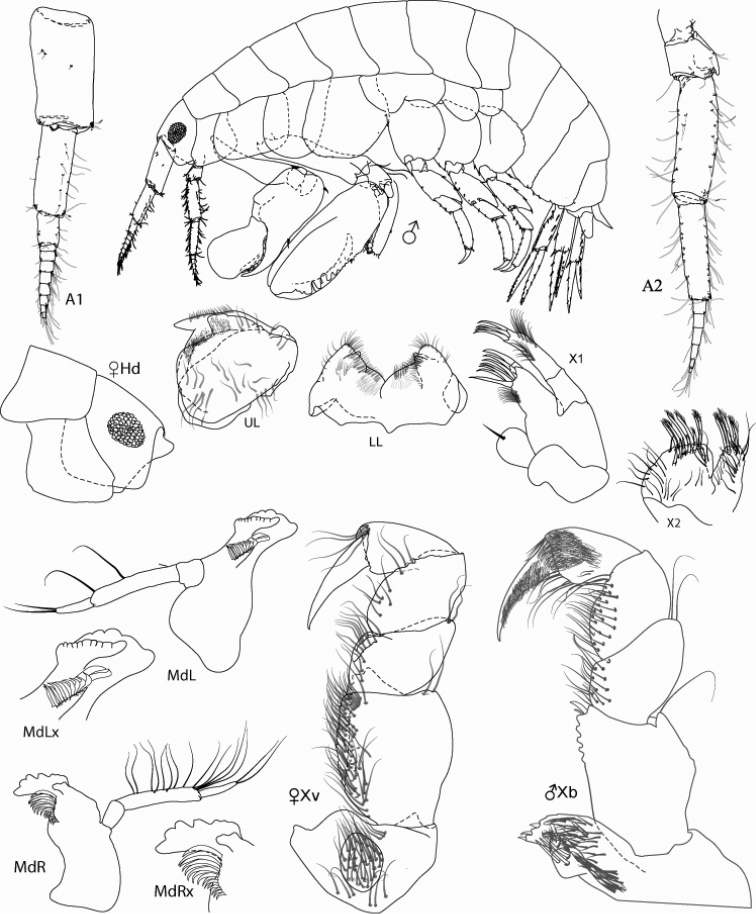
*Leucothoe eltoni* sp. n., holotype ♂A, 8.10 mm; paratype ♀B, 7.35 mm.

**Figure 2.**
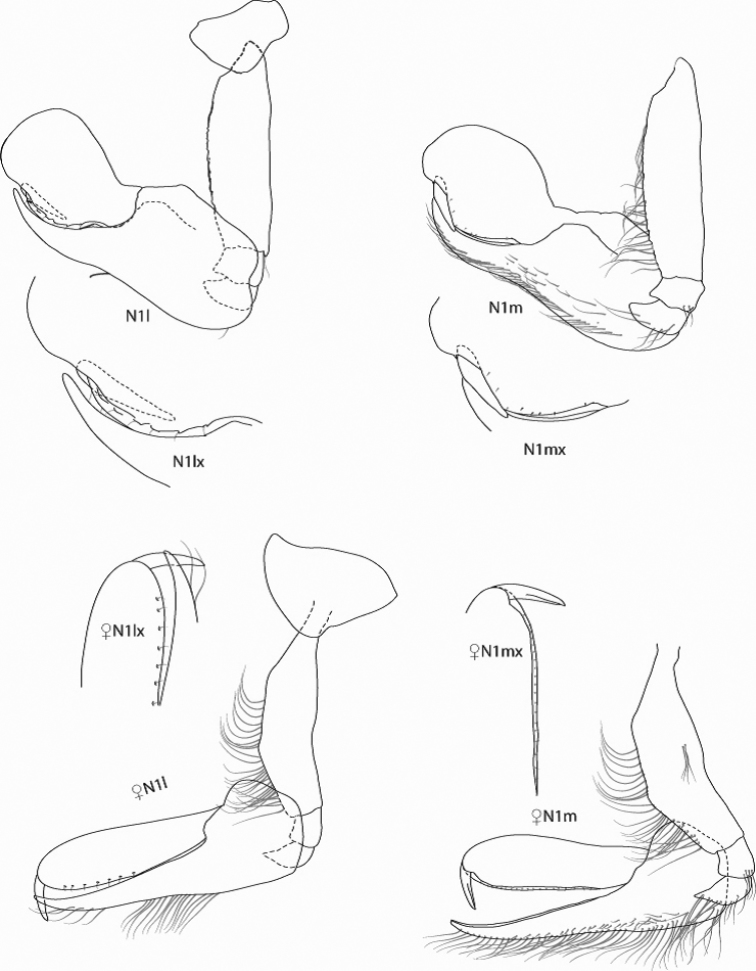
*Leucothoe eltoni* sp. n., gnathopod 1 lateral and medial; holotype ♂A, 8.10 mm; paratype ♀B, 7.35 mm.

**Figure 3.**
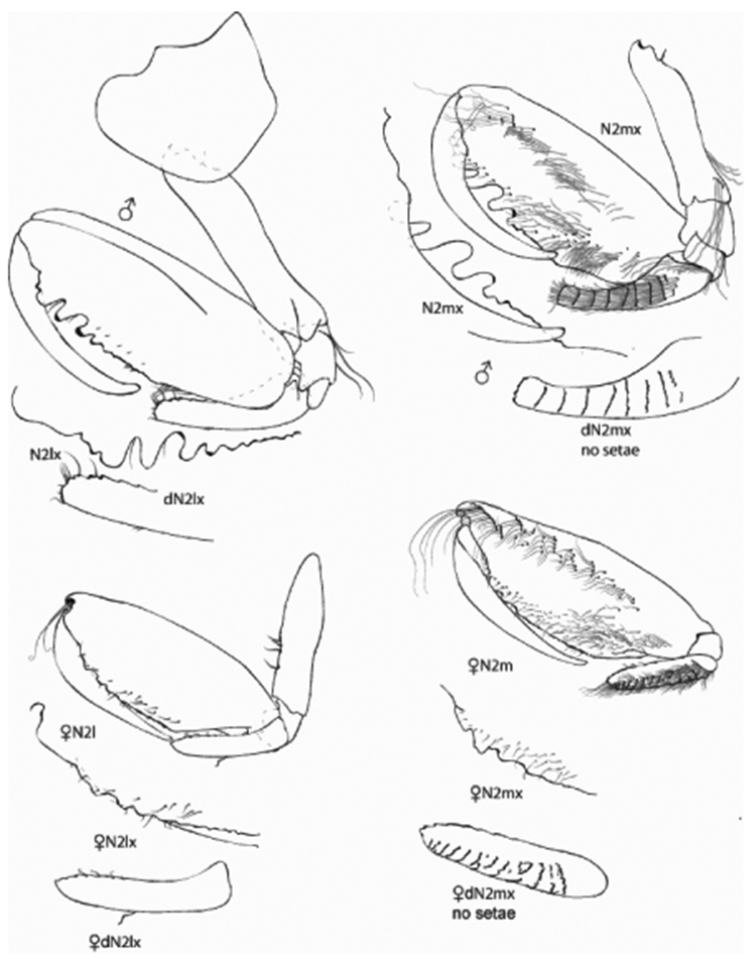
*Leucothoe eltoni* sp. n., gnathopod 2 medial and lateral; holotype ♂A, 8.10 mm; paratype ♀B, 7.35 mm.

**Figure 4.**
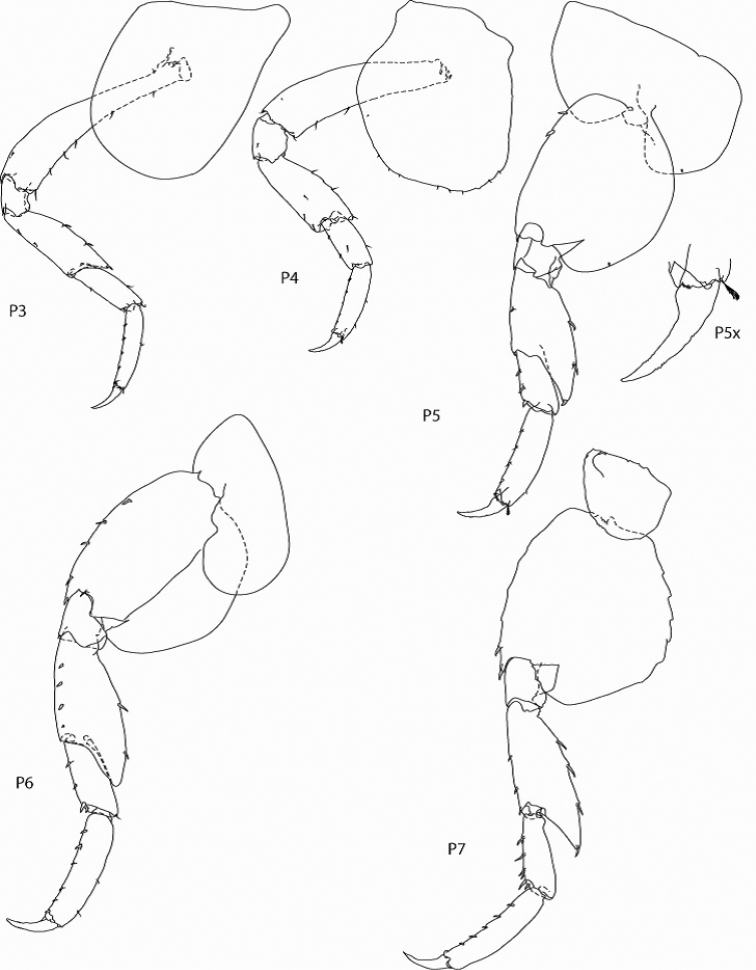
*Leucothoe eltoni* sp. n., holotype ♂A, 8.10 mm; pereopods 3-7.

**Figure 5.**
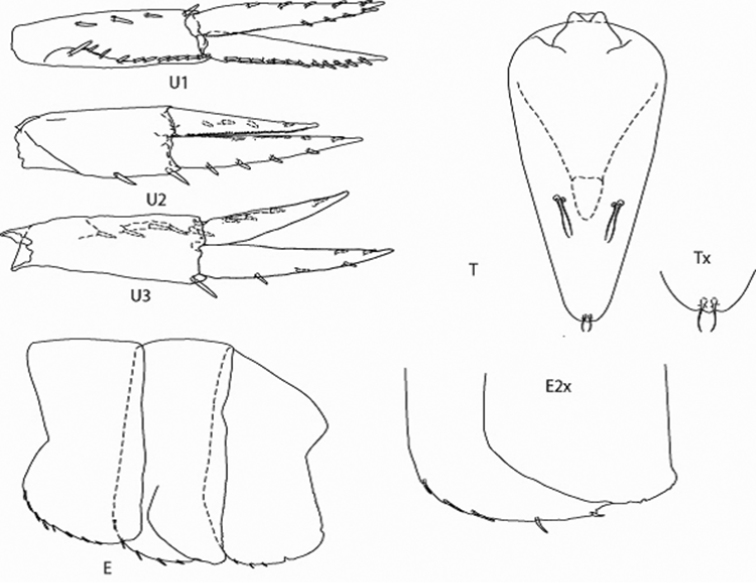
*Leucothoe eltoni* sp. n., holotype ♂A, 8.10 mm; uropods 1-3; epimera, telson.

**Figure 6.**
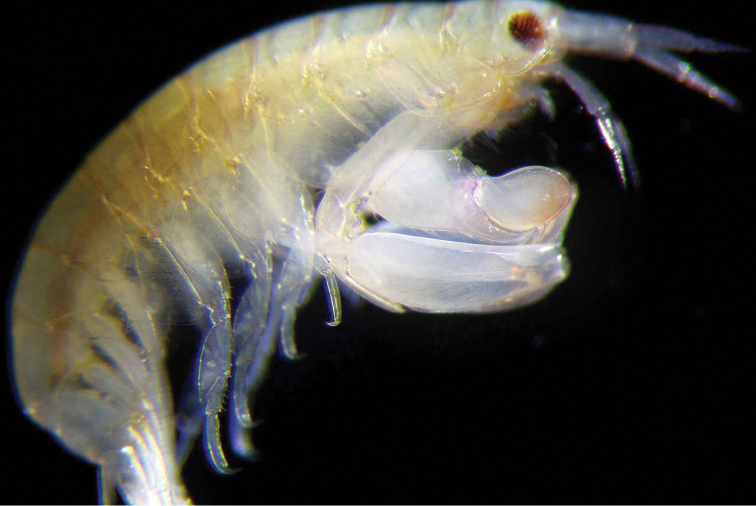
*Leucothoe eltoni* sp. n., male, whole body showing inflated gnathopod1. Photo J. Thomas.

**Figure 7.**
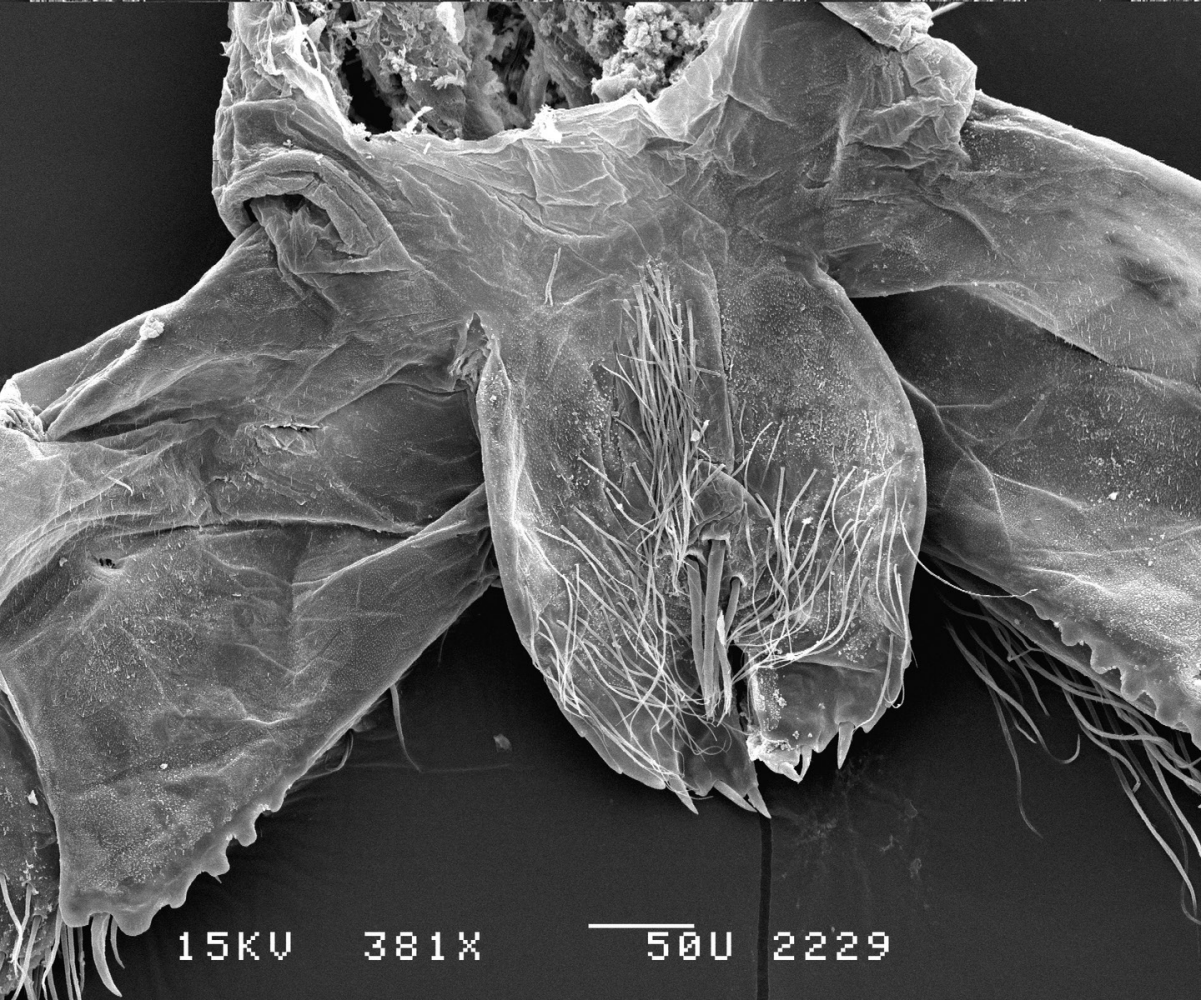
*Leucothoe eltoni* sp. n., SEM, male, maxilliped, 381×, Station Indo04-01c, Sulawesi, Indonesia.

**Figure 8.**
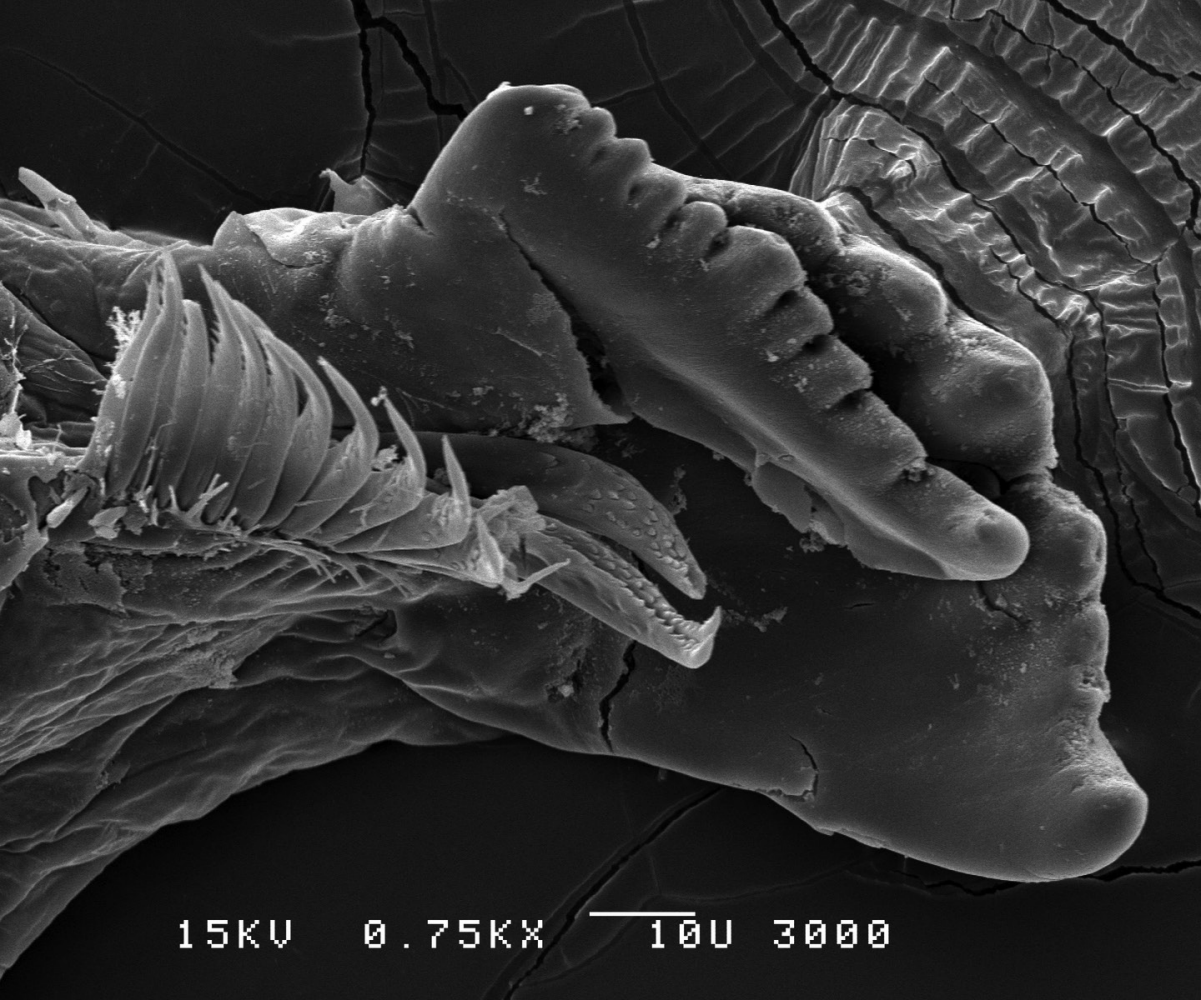
*Leucothoe eltoni* sp. n., SEM, male, left mandible, 400×; Station Indo04-01c, Sulawesi, Indonesia.

**Figure 9.**
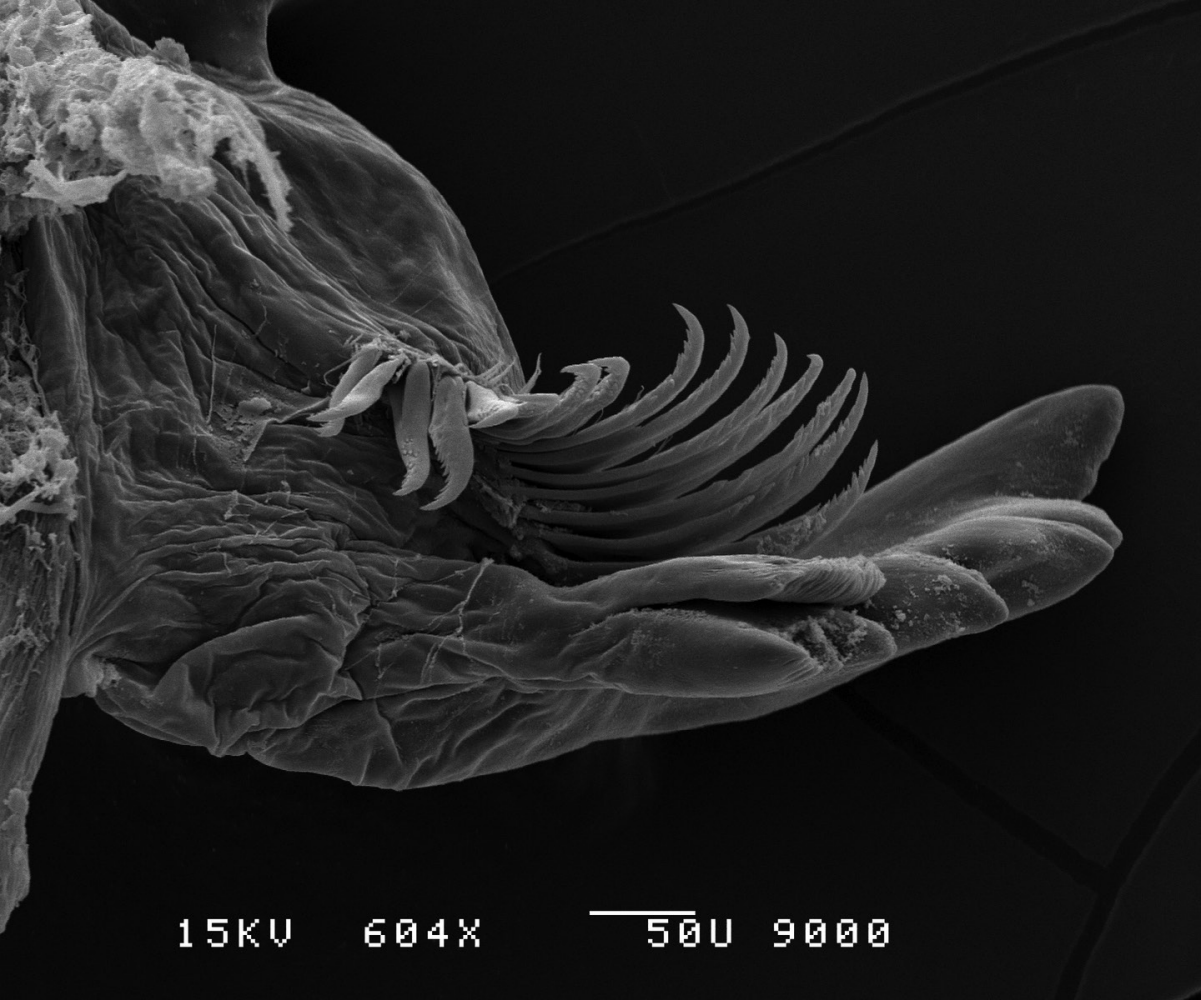
*Leucothoe eltoni* sp. n., SEM, male, right mandible, 600×; Station Indo04-01c, Sulawesi, Indonesia.

#### Relationships.

*Leucothoe eltoni* sp. n. most closely resembles *Leucothoe tumida* of Myers (2013) in the inflated carpus and propodus of gnathopod 1; in the short stubby antennae; pereopods 5-7 with article 4 extending more than 50 percent along posterior margin of article 5; and a 2-segmented maxilliped palp. *Leucothoe tumida* differs from *Leucothoe eltoni* in having a large excavation in the palm of gnathopod 2; and in having a smooth inner margin of the maxilliped outer plate. Both species differ in host preferences with *Leucothoe tumida* found in the mantle cavity of the winged pearl oyster *Pteria penguin* while *Leucothoe eltoni* prefers branchial chambers of large solitary ascidians, especially *Herdmania* and *Polycarpa* species.

Both *Leucothoe tumida* and *Leucothoe eltoni* superficially resemble members of the genus *Paraleucothoe* in the large inflated gnathopod 1 of terminal males. However, *Paraleucothoe* differs from all species of *Leucothoe* in having the outer plate of the maxilliped extended distally beyond palp article 1. *Paraleucothoe novaehollandiae* (Haswell, 1879) also has a uniarticulate maxilla 1 palp but this feature is no longer unique to the genus as a number of recently described *Leucothoe* species have this feature. *Paraleucothoe novaehollandiae* is reported from the branchial chambers of the stalked tunicates *Pyura spinifera* and *Pyura praeputialis* (formerly *Pyura stolonifera*) in southern Australia waters (Lowry et al 2000) and other large solitary tunicates such as *Herdmania* sp. The exact placement of *Paraleucothoe flindersi* described by Stebbing (1888) from the Torres Straits remains problematic as it lacks the extended apical lobe of maxilliped outer plate typical of *Leucothoe novaehollandiae*, but has a uniarticulate palp and gnathopod 1 reminiscent of *Leucothoe eltoni* females and juvenile males. Further resolution awaits examination of material from the type locality.

#### Ecology.

Coral reef**s**, coral rubble, found primarily in branchial baskets of solitary tunicates such as *Herdmania* and *Polycarpa* sp., rarely in bivalve mollusks (winged pearl oyster *Pteria penguin*), and branched yellow rope sponges *Callyspongia* (species undetermined).

#### Distribution.

Indonesia: Celebes Sea, Sulawesi, Kri Island, Halmera Sea, Raja Ampat Islands. Philippines: Cape Verde Passage, Mabini Tingloy. Hawaiian Islands (invasive): Ohau to Molokai, 2–20m.

#### Discussion.

While the native range of *Leucothoe eltoni* sp. n. encompasses shallow coral reef habitats in Indonesian and the Philippines, it is also an established invasive in Hawaiian waters (Coles et al. 1999). The most likely vector for introduction was a dry dock, USS *Machinist*, transported to Pearl Harbor from Subic Bay, Philippines in 1992. Prior extensive treatment of Hawaiian amphipods by J.L. Barnard (1970, 1971) and ongoing monitoring by the Bishop Museum did not document any leucothoid resembling *Leucothoe eltoni* sp. n. prior to 1992. Ongoing sampling of marine flora and fauna by the Bishop Museum first reported this species in 1997 as *Paraleucothoe flindersi*. Muir (1997) speculated it was most likely an introduced species. Such rafting on floating metal objects is a possible means of transportation for benthic marine organism (Cairns 2000; Creed and de Paula 2007, Hoeksema et al. 2012). Since first reported from Pearl Harbor in 1997 (as *Paraleucothoe flindersi*) *Leucothoe eltoni* sp. n. has spread throughout Oahu and other islands including Molokai. The author has collected *Leucothoe eltoni* sp. n. from sponges in Kaneohe Bay, Ohau. The effects, if any, of this species on endemic leucothoid commensals and its spread in Hawaii is unknown at this time.

## Supplementary Material

XML Treatment for Leucothoe eltoni
